# Ensemble‐based label adjudication (EBLA): A method for validating continuous physiological measures against observer‐dependent reference standards

**DOI:** 10.14814/phy2.71127

**Published:** 2026-10-09

**Authors:** Guillermo Gutierrez, Fernando Papa

**Affiliations:** ^1^ The George Washington University Washington DC USA; ^2^ Hitss Argentina Buenos Aires Argentina

**Keywords:** diagnostic accuracy, dyspnea, ensemble learning, label noise, mechanical ventilation

## Abstract

Comparing a novel physiological measure against an observer‐dependent reference standard creates uncertainty when results disagree because errors may arise from either method. We propose ensemble‐based label adjudication (EBLA), a computational framework that uses agreement between two imperfect instruments to create a high‐confidence training set for adjudicating discordant observations. EBLA was validated using the Wisconsin Breast Cancer Dataset (*n* = 569) with simulated label noise (10%–30%). Five classifiers were trained on concordant cases using stratified 5‐fold cross‐validation. In the primary 15%/15% noise scenario, majority‐vote EBLA improved accuracy from 0.85 to 0.97, sensitivity from 0.87 to 0.98, and specificity from 0.84 to 0.97. Supermajority and unanimity thresholds did not improve accuracy and excluded 1.4% and 2.5% of cases. EBLA consistently outperformed Cleanlab across all noise conditions. Application to 281 paired Mechanical Ventilation Respiratory Distress Observation Scale (MV‐RDOS) assessments and inspiratory effort measurements (PmusPTP) from 45 mechanically ventilated patients increased accuracy from 0.81 to 0.86, primarily by improving specificity from 0.88 to 0.94. EBLA provides a transparent, interpretable framework for sensitivity analysis when validating physiological measurements against imperfect observer‐dependent reference standards.

## Graphical abstract

Ensemble‐Based Label Adjudication for validating continuous physiological measures against observer‐dependent reference standards.

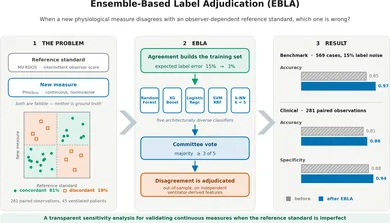

## 1 INTRODUCTION

Dyspnea affects approximately 50% of mechanically ventilated (MV) patients and is associated with prolonged ventilation, increased mortality, and post‐traumatic stress disorder (Demoule et al., 2022; Worsham et al., 2021). Because dyspnea is a subjective symptom, its detection in non‐communicative patients depends on behavioral observation tools. The Mechanical Ventilation Respiratory Distress Observation Scale (MV‐RDOS) is the most extensively validated instrument for this purpose (Decavèle et al., 2018), but it is observer‐dependent, applied intermittently, and susceptible to inter‐rater variability. As such, it represents an imperfect, or noisy, reference standard.

We recently reported that the pressure–time product of inspiratory effort (PmusPTP), estimated continuously and noninvasively from ventilator‐derived airway pressure and flow signals (Gutierrez, 2022; Gutierrez, 2024), correlates with MV‐RDOS scores in a prospective two‐center study (Gutierrez et al., 2026). At a threshold of 20 cmH_2_O·s·min^−1^, PmusPTP identified dyspnea with an area under the receiver‐operating characteristic curve (AUC) of 0.74. However, nearly one‐fifth of paired observations were discordant, raising a fundamental question: when a novel physiological measure disagrees with an imperfect reference standard, which method is wrong?

This problem extends well beyond dyspnea monitoring and is common whenever new physiological measurements are validated against observer‐dependent assessments. Conventional diagnostic accuracy analyses assume that reference labels are correct; however, performance estimates may be systematically biased when this assumption is violated (Whiting et al., 2011). Existing label‐correction methods, such as confident learning (Cleanlab) (Frénay & Verleysen, 2014; Northcutt et al., 2021), typically operate on a single noisy label source and therefore require one source to be designated as the reference. They do not exploit the information contained in agreement and disagreement between two independently fallible instruments.

To address this limitation, we developed Ensemble‐Based Label Adjudication (EBLA), a deliberately symmetric framework that treats both measurements as fallible and privileges neither as correct. EBLA exploits the statistical enrichment of correct labels among observations where two independent instruments agree. These concordant observations form a high‐confidence training set from which an ensemble of machine‐learning classifiers learns to adjudicate discordant cases. EBLA is intended to assess the potential impact of reference‐standard error on validation results, not to replace the primary analysis. Importantly, the patient's underlying dyspnea state cannot be directly observed in non‐communicative patients; neither MV‐RDOS nor PmusPTP can therefore be considered ground truth. Throughout this manuscript, “reference standard” denotes the conventional comparator, not an assumed error‐free standard.

Because the clinical dyspnea dataset lacks an independent ground truth, we first evaluated EBLA using the Wisconsin Breast Cancer Dataset (Wolberg et al., 1995). This dataset contains pathology‐confirmed diagnoses that permit objective assessment of performance under simulated label noise. We then applied EBLA to the clinical MV‐RDOS dyspnea dataset and examined the physiological implications of concordant and discordant observations. The two datasets are clinically unrelated: the breast‐cancer data serve solely as a controlled methodological testbed with known ground truth, allowing us to quantify how accurately EBLA recovers correct labels before the method is applied to the dyspnea problem, where no ground truth exists.

## 2 METHODS

### 2.1 Overview of EBLA

Figure 1 illustrates the EBLA workflow. Paired observations from a novel method and an imperfect reference standard are first classified into the four quadrants of a confusion matrix using predefined thresholds (Figure 1a). The central premise of EBLA is that concordant observations, those classified as either putative true positives or putative true negatives (shown as green dots), are enriched for correctly labeled cases and therefore have a substantially lower probability of label error than the dataset as a whole. If each method has an independent error rate *ε*, the expected error rate among concordant pairs is $\varepsilon^{2}/\left[{\left(1-\varepsilon \right)}^{2}+\varepsilon^{2}\right]$. Thus, for ε=15%, the expected error rate falls to approximately 3.0% among concordant observations, a fivefold reduction in label contamination (See note on expected label contamination in Data S1).

**FIGURE 1:**
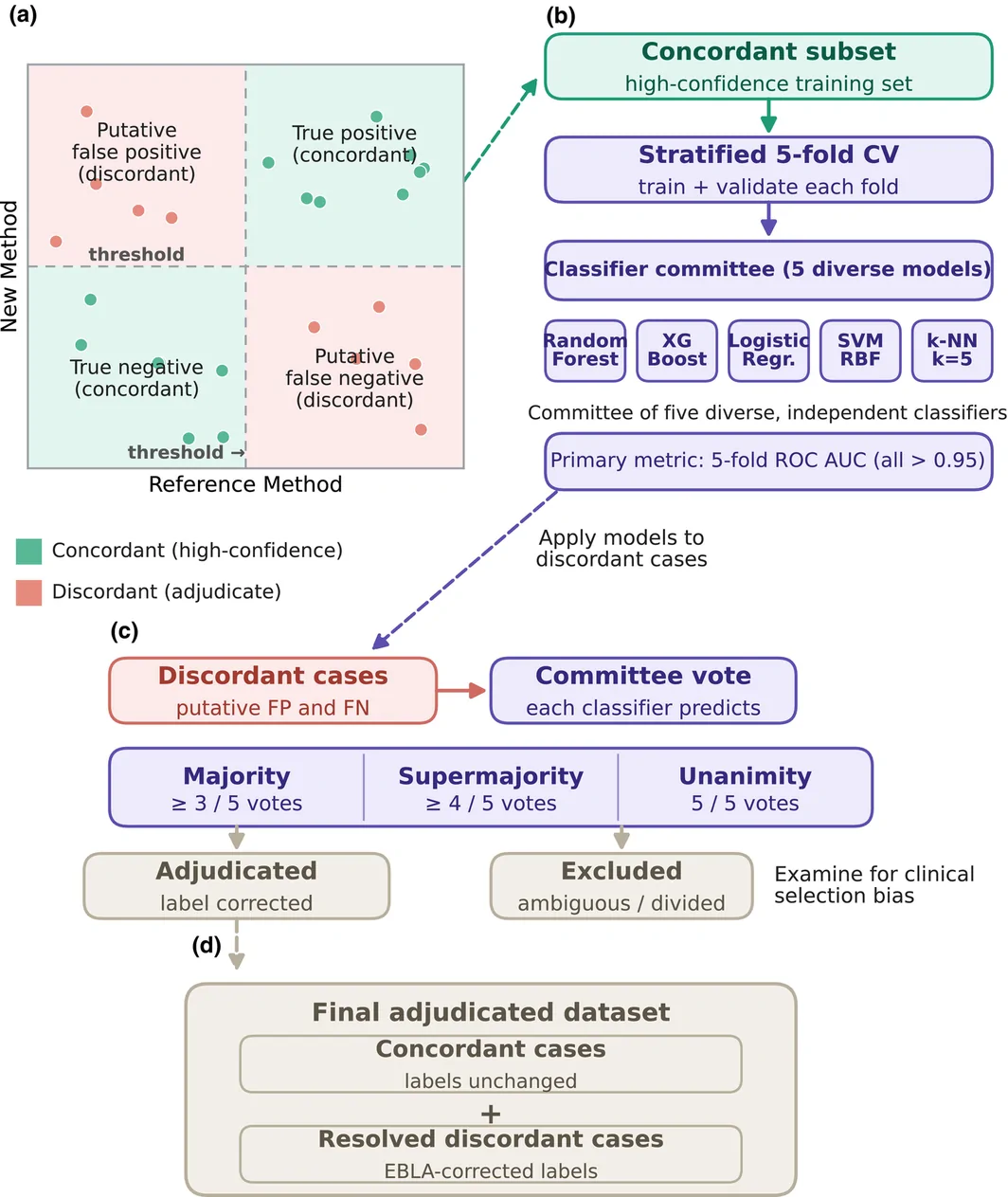
EBLA workflow overview. (a) Paired observations from a new measurement method and an imperfect reference standard are plotted and partitioned into four quadrants using predefined method‐specific thresholds, yielding concordant (green: True positives and true negatives) and discordant (red: Putative false positives and false negatives) observations. (b) The concordant observations, which are enriched for correct labels, form a high‐confidence training set used to train a committee of five diverse classifiers using stratified 5‐fold cross‐validation. (c) The trained committee adjudicates each discordant observation by threshold voting. The majority rule (≥3 of 5 votes) is illustrated, although supermajority (≥4 of 5) and unanimity (5 of 5) thresholds may also be used. (d) Under majority voting, the final dataset consists of all concordant observations together with all adjudicated discordant observations. Under supermajority or unanimity voting, discordant observations that fail to meet the selected voting threshold are designated ambiguous and reported separately.

This enriched concordant subset is used to train a committee of five diverse machine‐learning (ML) classifiers (Figure 1b): Random Forest (100 trees), XGBoost, Logistic Regression (maximum iterations = 1000), Support Vector Machine with a radial basis function kernel, and *k*‐Nearest Neighbors (*k* = 5). Classifier diversity is a fundamental design feature because adjudication of discordant cases relies on independent decision‐making across models. Accordingly, architecturally similar algorithms are deliberately excluded to minimize correlated errors and maximize the value of ensemble voting. The five learners were chosen to span distinct inductive biases: linear (logistic regression), kernel (support vector machine), bagged trees (random forest), gradient boosting (XGBoost), and instance‐based (*k*‐nearest neighbors), thereby minimizing correlation among their errors.

The trained classifier committee is then applied to discordant observations using threshold‐based voting (Figure 1c). Discordant observations are adjudicated according to a chosen decision threshold: majority (≥3 of 5 votes), supermajority (≥4 of 5 votes), and unanimity (5 of 5 votes). For majority decision threshold, the odd number of classifiers allows for binary decision making and all cases are adjudicated. For supermajority or unanimity decision thresholds, cases that fail to achieve the level of agreement are designated ambiguous and excluded from adjudication. The final dataset (Figure 1d) comprises all concordant observations plus the EBLA resolved discordant cases. Ambiguous cases, if any, are retained and reported separately.

### 2.2 Conditions for EBLA applicability

EBLA is most appropriate when the following conditions are satisfied: (1) errors between the two instruments are at least partially independent, since correlated errors (e.g., both methods being influenced by deep sedation) reduce the purity of the concordant set; (2) the concordant subset is sufficiently large to support reliable classifier training (in our experience, at least 80 concordant observations), with class imbalance evaluated and addressed as needed; (3) informative features are available for classifier training that are independent of those used to define the classification thresholds; and (4) the voting threshold is specified a priori according to the relative consequences of false‐positive and false‐negative classifications.

### 2.3 EBLA method validation

We selected the Wisconsin Breast Cancer Dataset for methodological validation of EBLA because it permits objective quantification of label recovery, is publicly available and widely used in label‐correction benchmarking, and is free of assumptions regarding the behavior of clinical diagnostic instruments. The Wisconsin Breast Cancer Dataset (Wolberg et al., 1995) comprises 569 cases (212 malignant, 357 benign; 37.3% prevalence) characterized by 30 continuous features derived from digitized fine‐needle aspiration images (See Data S1). Pathology‐confirmed diagnoses served as the ground‐truth reference standard.

To create a controlled validation environment, we simulated a scenario in which both a reference standard and a new measurement are imperfect and may misclassify observations independently. Because the true labels are known, the accuracy of EBLA in recovering mislabeled observations can be quantified directly.

Two noisy versions of the true dataset labels, one representing the reference standard and one the new method, were then generated by randomly flipping class assignments at predefined error rates. Only the class labels were perturbed; the continuous feature values were left unchanged. Label corruption was applied separately to each label set using distinct random streams initialized with a fixed random seed (42) for repeatability.

Five error‐rate combinations were evaluated: (10%, 10%), (15%, 15%), (20%, 15%), (20%, 20%), and (30%, 20%), representing both symmetric and asymmetric noise conditions. The 15%/15% combination was designated the primary scenario because it approximates both the roughly 19% discordance observed in the clinical cohort and a plausible error range for behavioral scales such as MV‐RDOS; the remaining scenarios assess robustness to higher and asymmetric noise. We recognize that real‐world measurement errors are often systematic rather than purely random; this limitation is discussed below.

The five ML classifiers were trained on the concordant subset and classifier performance was evaluated using stratified 5‐fold cross‐validation, with area under the receiver‐operating characteristic curve (ROC AUC) as the primary metric.

### 2.4 Cross‐validation

The cross‐validation procedure was designed to provide an unbiased estimate of classifier performance. The concordant subset was partitioned into five stratified folds, preserving the proportion of positive and negative cases within each fold. During each iteration, four folds were used for training and the remaining fold served as the test set. Each classifier was trained on the training folds and evaluated on the held‐out fold, with performance summarized by the area under the receiver‐operating characteristic curve (ROC AUC). The reported ROC AUC represents the average across all five test folds.

Stratification was used to mitigate the effects of class imbalance. To prevent information leakage, feature standardization (zero mean, unit variance) was performed separately within each cross‐validation iteration using scaling parameters estimated from the training folds only. These parameters were then applied to the corresponding test fold. This approach ensures that the test data remain completely unseen during preprocessing and avoids the optimistic bias that would result from fitting the scaler before data partitioning.

Following cross‐validation, each classifier was retrained on the full concordant dataset using feature‐standardization parameters derived from that same dataset. This step maximizes the amount of labeled data available for learning the decision boundary before deployment for adjudication. The discordant observations were excluded from all stages of model development, including classifier training, cross‐validation, feature scaling, and threshold selection. They were used solely as a held‐out prediction set for adjudication. Consequently, the classification of discordant cases represents true out‐of‐sample prediction rather than in‐sample relabeling.

### 2.5 Clinical application

The clinical utility of EBLA was evaluated using a publicly available MV‐RDOS Dyspnea Dataset consisting of 281 paired MV‐RDOS–PmusPTP observations obtained from 45 noncommunicative mechanically ventilated patients enrolled at two academic centers (Gutierrez et al., 2026). Discordant observations were defined as MV‐RDOS ≥2.6 with PmusPTP <20 cmH_2_O·s·min^−1^ (putative false negatives) or MV‐RDOS <2.6 with PmusPTP ≥20 cmH_2_O·s·min^−1^ (putative false positives).

Classifier training features included peak airway pressure, its coefficient of variation, respiratory system compliance, driving pressure, respiratory rate, tidal volume, and the respiratory variability index (Gutierrez et al., 2011). Features were standardized using parameters derived from the concordant subset. The same classifier committee and majority voting threshold used in the Wisconsin Breast Cancer Dataset analysis were then applied to adjudicate discordant observations.

### 2.6 Performance evaluation

Performance was assessed using accuracy, sensitivity, and specificity calculated against the true labels in the simulation study and against the adjudicated reference in the clinical application. Metrics were computed before and after EBLA adjudication to quantify the impact of label correction.

#### 2.6.1 Independent comparator

As an independent benchmark, the confident‐learning method Cleanlab (9) was applied to the same noisy labels used to train the committee. Cleanlab identifies likely mislabeled instances by comparing observed labels with out‐of‐sample predicted class probabilities. Flagged cases had their noisy labels inverted.

#### 2.6.2 Software

Analyses were performed in Python 3.11 using scikit‐learn (Pedregosa et al., 2011), XGBoost (Chen & Guestrin, 2016), the open‐source Cleanlab, NumPy, and pandas. Analysis code and datasets are provided in the Data S1 and public repositories (see Data Availability Statement).

## 3 RESULTS

### 3.1 Dataset composition and label enrichment

For the Wisconsin Breast Cancer Dataset primary 15%/15% scenario, 429 cases (75.4%) were concordant and 140 (24.6%) discordant. As predicted by the independence assumption, label contamination was reduced from an empirically observed 27.8% in the full dataset to 3.0% in the conco‐fold enrichment. This concordance proportion closely mirrors the 81% concordance observed in the clinical MV‐dyspnea cohort (see below), supporting the realism of the simulation.

### 3.2 Classifier performance

All five classifiers achieved high cross‐validated performance on the concordant subset (Table 1). AUC ranged from 0.953 (*k*‐NN) to 0.966 (Logistic Regression) in the primary scenario, confirming that the enriched labels retained strong discriminative signal and that classifier diversity was maintained without compromising individual accuracy.

**TABLE 1:** Classifier performance on the concordant data subset (15%/15% noise scenario).

Classifier	Cross‐validated AUC (mean ± SD, 95% CI)	Architecture type
Random Forest	0.960 ± 0.020 (0.942, 0.978)	Ensemble tree
XGBoost	0.957 ± 0.022 (0.938, 0.976)	Gradient boosting
Logistic Regression	0.966 ± 0.018 (0.950, 0.982)	Linear
Support Vector Machine	0.961 ± 0.019 (0.944, 0.978)	Kernel (RBF)
*k*‐Nearest Neighbors	0.953 ± 0.024 (0.932, 0.974)	Instance‐based

Abbreviations: AUC, area under the receiver‐operating characteristic curve; CI, 95% confidence interval; SD, standard deviation.

### 3.3 EBLA performance across noise scenarios

In the primary scenario (15%/15%), EBLA with a majority threshold (≥3/5) improved accuracy from 0.85 to 0.97 (Figure 2). Even in the most demanding condition (30%/20%), accuracy improved from 0.80 to 0.94. EBLA outperformed Cleanlab across all scenarios. The performance advantage increased with noise level (absolute accuracy gain over Cleanlab: 0.01 at 10%/10% to 0.04 at 30%/20%), reflecting EBLA's protection against noise propagation during training: Cleanlab trains on the full noisy dataset and corrects post hoc, whereas EBLA trains exclusively on the enriched concordant subset.

**FIGURE 2:**
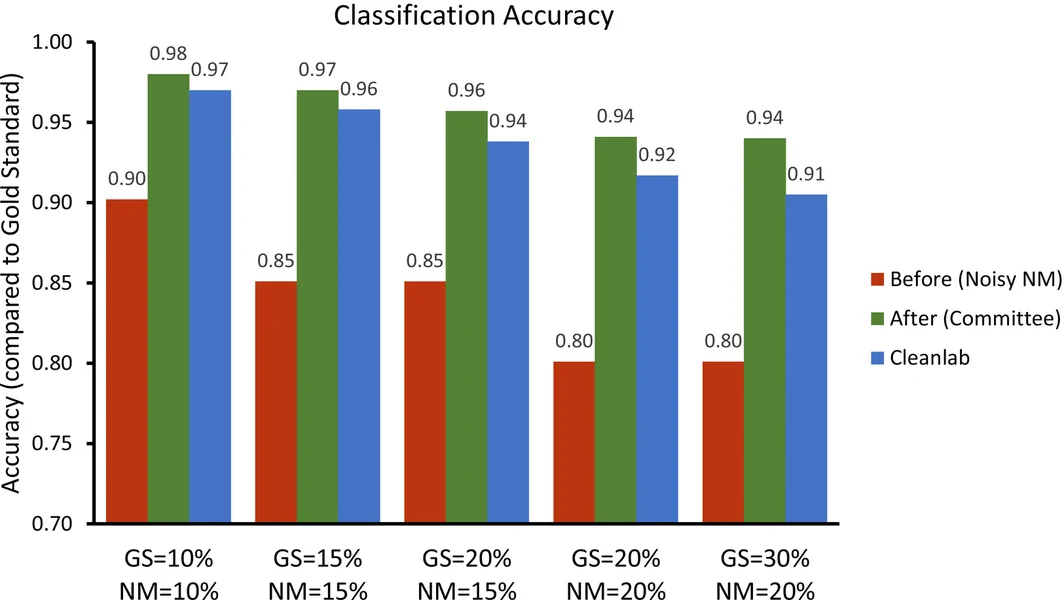
Method validation. Accuracy improvement across five noise‐rate scenarios using majority threshold (≥3/5). Grouped bar chart comparing accuracy before adjudication (red), after EBLA adjudication (green), and after Cleanlab correction (blue). Numeric values are annotated above bars. GS, simulated gold standard error rate; NM, simulated new method error rate. All values computed against true pathology labels.

As shown in Table 2, performance gains were consistent for all scenarios. For the primary scenario (15%/15%), sensitivity improved from 0.87 to 0.98, and specificity improved from 0.84 to 0.97, with no cases excluded as ambiguous. The majority threshold provided the best balance between accuracy and data retention (Table 3). Supermajority and unanimity yielded marginally higher accuracy but excluded more cases, whereas the majority voting threshold retained all cases with similar performance.

**TABLE 2:** Sensitivity and specificity before and after EBLA adjudication using the majority threshold (≥3/5) across five noise scenarios (*n* = 569 cases).

Scenario (GS/NM)	Concordant	Discordant	Sensitivity before	Sensitivity after	Specificity before	Specificity after
10%/10%	469	100	0.877	0.967	0.916	0.992
15%/15%	429	140	0.868	0.976	0.840	0.966
20%/15%	411	158	0.849	0.948	0.852	0.960
20%/20%	399	170	0.835	0.948	0.782	0.935
30%/20%	350	216	0.788	0.943	0.810	0.938

*Note*: All metrics computed against true pathology labels.

Abbreviations: GS, simulated gold standard error rate; NM, simulated new method error rate.

**TABLE 3:** Comparison of sensitivity and specificity after EBLA adjudication using majority (≥3/5), supermajority (≥4/5), and unanimity (5/5) thresholds across noise scenarios (*n* = 569 cases).

Scenario (GS/NM)	Sensitivity			Specificity			Excluded^a^	
	Majority	Super‐majority	Unanimity	Majority	Super‐majority	Unanimity	Super‐majority	Unanimity
10%/10%	0.967	0.971	0.976	0.992	0.992	0.992	3	9
15%/15%	0.976	0.976	0.976	0.964	0.966	0.965	8	14
20%/15%	0.948	0.952	0.956	0.961	0.960	0.962	7	19
20%/20%	0.948	0.953	0.957	0.933	0.935	0.936	5	15
30%/20%	0.943	0.943	0.946	0.936	0.938	0.937	4	16

*Note*: All metrics computed against true pathology labels.

Abbreviations: GS, simulated gold standard error rate; NM, simulated new method error rate.

^a^ No excluded cases with majority threshold.

### 3.4 The MV‐RDOS‐PmusPTP dyspnea dataset

The clinical motivation for EBLA was validation of continuously measured P_mus_PTP as a marker of dyspnea by comparison to intermittent MV‐RDOS assessments. The MV‐RDOS‐PmusPTP dataset contains 281 data pairs obtained from 45 non‐communicative mechanically ventilated patients. When compared to MV‐RDOS ≥2.6 dyspnea threshold, values of P_mus_PTP ≥ 20 cmH_2_O·s·min^−1^ discriminated dyspnea with an AUC of 0.74. The comparisons yielded 227 concordant observations (80.8%; 189 TN, 38 TP) and 54 discordant (19.2%; 27 FP, 27 FN) (Figure 3a).

**FIGURE 3:**
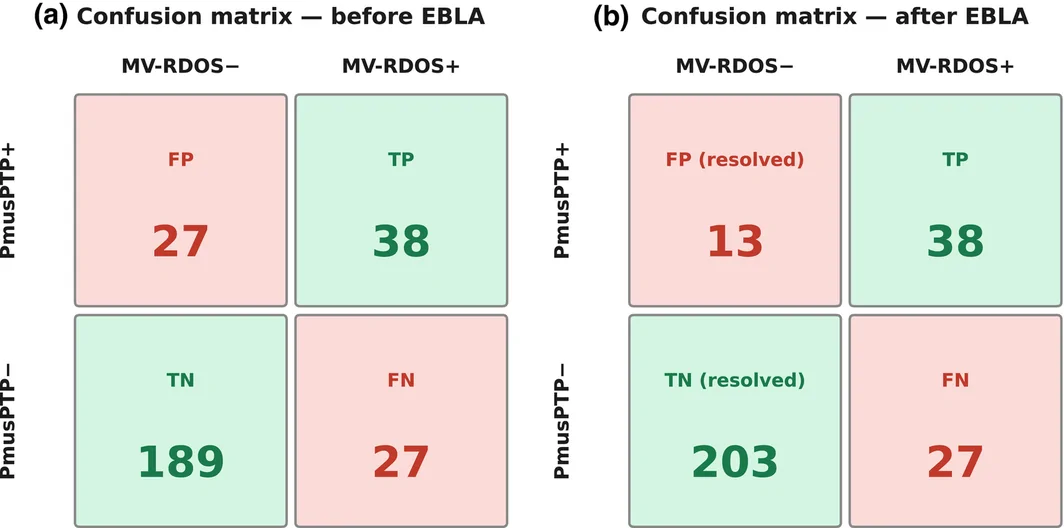
Clinical application. MV‐dyspnea dataset. (a) The 2 × 2 confusion matrix prior to EBLA, with the 281 observations broken into TP = 38, FP = 27, TN = 189, FN = 27. Concordant (*n* = 227, 80.8%) and discordant (*n* = 54, 19.2%) totals. (b) Post‐EBLA confusion matrix showing the resolved discordant cases: FP drops from 27 to 13 (resolved TN), while FN remains at 27. Accuracy increases from 81% to 86% and specificity rises from 0.88 to 0.94 while sensitivity holds at 0.59.

Application of EBLA to the concordant cases resulted in classifier AUCs ranging from 0.923 to 0.969 with peak pressure and its coefficient of variation being the most important features (Figures S1 and S2; Data S1). The 54 discordant cases were resolved after classifier adjudication and majority committee voting threshold (Figure 3b). Putative false positives were substantially reduced from 27 to 13, while putative false negatives remained unchanged. Overall P_mus_PTP accuracy improved from 81% to 86%, driven by a marked increase in specificity from 88% to 94% with no improvement in sensitivity (59%).

## 4 DISCUSSION

### 4.1 Learning from imperfect reference standards

EBLA addresses a long‐recognized problem in diagnostic medicine: evaluating a measurement against a fallible reference standard. Hui and Walter (Liu et al., 2023) showed that treating an imperfect reference as error‐free can bias estimates of diagnostic accuracy and proposed joint estimation of test performance when two conditionally independent tests are applied in populations with differing disease prevalence. Other approaches to the “no gold standard” problem include correction for reference‐standard error, composite or consensus standards, latent‐class models, and validation against clinical follow‐up or outcomes (Hui & Walter, 1980; Reitsma et al., 2009; Rutjes et al., 2007).

In parallel, the machine‐learning literature has developed methods for learning from noisy or misclassified labels (Sun & Zhou, 2025). EBLA connects these concepts by combining agreement‐based label enrichment with machine‐learning adjudication. When two informative measurements have sufficiently independent error processes, concordant observations are enriched for correct classifications because simultaneous matching errors are less frequent than matching correct classifications. EBLA uses these concordant observations to train five structurally different classifiers using features not used to define the original agreement. The resulting ensemble is then applied only to discordant observations, with the final classification determined by committee vote. Thus, neither measurement is assumed to be error‐free. This design rests on a classical result in ensemble learning: a majority vote among accurate classifiers is more reliable than any individual member precisely when the members are diverse, that is, when they do not make the same errors, so that correct votes outnumber coincident mistakes (Dietterich, 2000).

The Wisconsin benchmark demonstrated that this strategy improves label recovery over an established confident‐learning approach across a range of simulated noise conditions. Application to the clinical dyspnea dataset further showed that EBLA produces physiologically coherent adjudications, supporting its validity in a clinically relevant setting where no independent ground truth exists.

EBLA is broadly applicable to studies comparing two imperfect measurement methods, including imaging interpretation, clinical scoring systems, automated monitoring technologies, and diagnostic accuracy studies lacking a definitive reference standard. The framework requires only standard machine‐learning libraries, is computationally straightforward, and is readily scalable to datasets of moderate size.

EBLA should be viewed as a sensitivity‐analysis framework rather than a replacement for conventional validation. Its purpose is to estimate the robustness of diagnostic performance when the reference standard is imperfect by providing plausible bounds on performance rather than a single definitive estimate. Accordingly, EBLA does not alter or rescue the primary analysis. If the unadjusted analysis demonstrates poor discriminative performance, that conclusion remains unchanged. Instead, EBLA asks whether part of the observed disagreement reflects noise in the reference standard rather than limitations of the new measurement. Agreement between adjusted and unadjusted results strengthens confidence in the primary findings, whereas divergence identifies observations that warrant closer physiological and clinical investigation.

### 4.2 Potential for circularity

A potential concern is that EBLA is circular or merely reinforces agreement between the two instruments. This is not the case because the information used to identify discordant observations is distinct from the information used to adjudicate them.

Protection against circularity rests on two structural properties of the method. First, concordance is defined solely by the threshold classifications of the two instruments. In the MV‐RDOS–PmusPTP application, these thresholds are based exclusively on MV‐RDOS and PmusPTP. In contrast, classifier training uses a separate set of physiological and ventilator‐derived features; neither the concordance label nor the threshold‐defining variables are provided as classifier inputs. Consequently, adjudication is based on an independent feature space rather than on the variables that created the discordance.

Second, EBLA relies on a committee of architecturally diverse classifiers, including linear, kernel‐based, tree‐ensemble, gradient‐boosting, and instance‐based methods. Agreement among these models therefore reflects convergent evidence across fundamentally different decision boundaries rather than reinforcement of a shared modeling artifact.

### 4.3 Value of the classifier committee

A reasonable question is whether a committee is necessary when individual classifiers already achieve high cross‐validated AUC. Table S1 shows why selecting a single classifier on this basis is problematic. In the Wisconsin benchmark, committee performance remained close to the best individual classifier at lower noise levels and exceeded all individual models at the highest noise level (0.935 vs. ≤0.930). In the clinical cohort, logistic regression had the highest AUC (0.969; Table 1) but the lowest specificity and accuracy after adjudication (Table S1b), whereas SVM, with a lower AUC (0.928; Table 1), performed best. The majority committee provided intermediate performance without requiring advance selection of a particular model. The committee therefore reduces reliance on any single model‐selection decision. Mean pairwise disagreement among classifiers was 11.1%, indicating substantial but incomplete concordance. Thus, the rationale for the ensemble is robustness through consensus rather than an expectation that it will always outperform any individual classifier.

### 4.4 Rationale for selection of comparator

Cleanlab was selected as the comparator for the Wisconsin benchmark because pathology‐confirmed diagnoses provide a definitive ground truth, allowing objective quantification of label recovery. In this setting, Cleanlab's case‐level label correction can be evaluated directly against verified diagnoses.

Cleanlab identifies likely mislabeled observations using out‐of‐sample predicted class probabilities estimated from the entire dataset. Consequently, classifier learning is influenced by all mislabeled observations before any label correction is performed. In contrast, EBLA trains exclusively on the concordant subset, whose expected label error rate is $\varepsilon^{2}/\left[{\left(1-\varepsilon \right)}^{2}+\varepsilon^{2}\right]$ rather than 2*ε* − *ε* ^2^. The resulting improvement in the signal‐to‐noise ratio yields more reliable decision boundaries for adjudicating discordant observations. These conceptual differences become increasingly important as label noise increases. Consistent with this expectation, EBLA's advantage over Cleanlab widened with increasing noise, as illustrated in Figure 2 showing a gain in accuracy from 0.009 to 0.035 as label noise increases from 10%/10% to 30%/20%.

Cleanlab is appropriate for benchmarking against known ground truth but not for adjudicating disagreement between two independently imperfect clinical instruments. Accordingly, Cleanlab was not applied to the MV‐RDOS–PmusPTP dataset because no independent gold standard exists. Both MV‐RDOS and PmusPTP are imperfect indicators of an unobservable latent dyspnea state. Applying Cleanlab would require designating one instrument as the reference against which the other is corrected, thereby implicitly assuming that it is error‐free. This assumption contradicts the central premise of EBLA that neither instrument can be assumed to represent the truth.

### 4.5 Feature interpretability

EBLA also provides measures of feature importance. For the MV‐RDOS‐PmusPTP database, EBLA identified ventilator‐derived variables, including peak airway pressure and its coefficient of variation, as major contributors to adjudication. This transparency supports the physiological plausibility of the adjudications and may generate mechanistic hypotheses about the causes of discordance, informing both future validation studies and the design of monitoring systems.

### 4.6 Physiological interpretation of the MV‐dyspnea findings

The predominant improvement in specificity observed in the clinical application (88%–94%), with sensitivity remaining unchanged (59%), is physiologically plausible and reflects the direction in which EBLA adjudicated discordant observations.

Most of the specificity gain resulted from reclassifying 14 of the 27 putative false‐positive observations as true negatives. These cases exceeded the PmusPTP threshold but lacked the ventilator‐derived waveform patterns characteristic of sustained inspiratory effort. Such observations are consistent with transient or non‐sustained efforts, sighs, coughs, brief patient–ventilator asynchrony, or estimation noise. Because the ventilator signal morphology and the intermittent bedside assessment both suggested quiet breathing, the classifier committee identified these observations as more likely to represent overestimation by PmusPTP than missed dyspnea by MV‐RDOS. For continuous monitoring, this reduction in false positives could lessen alarm burden and unnecessary intervention.

In contrast, sensitivity did not improve because many putative false‐negative observations likely represent a different physiological mechanism. Dyspnea driven primarily by air hunger, anxiety, or other non‐mechanical factors may occur without a sustained increase in inspiratory effort and therefore produces little or no ventilator‐derived signature. Such observations are not identifiable from PmusPTP or the physiological features available to EBLA. Consequently, EBLA can refine classifications among mechanically expressed dyspnea states but cannot recover dyspnea that leaves no detectable mechanical trace.

### 4.7 Limitations

Several limitations should be considered. First, EBLA assumes that errors in the two instruments are at least partially independent. Correlated errors, for example, deep sedation simultaneously reducing respiratory effort and behavioral manifestations of dyspnea could decrease the purity of the concordant set and reduce the effectiveness of adjudication.

Second, the Wisconsin benchmark study simulated independent random label noise, whereas clinical measurement errors are often systematic, class‐dependent, and context‐specific. Accordingly, the benchmark evaluated the methodological performance of EBLA under controlled conditions rather than reproducing the full complexity of clinical error.

Third, as instrument error rates increase, the concordant training set becomes both smaller and more contaminated, reducing classifier performance. Assessment of concordant‐set size and purity is therefore recommended before applying EBLA.

Fourth, the clinical application involved a single exploratory dataset of 45 patients from two centers with heterogeneous populations, ventilator management, and sedation practices. Because only one MV‐RDOS assessment was obtained at each timepoint, inter‐rater variability could not be estimated. Prospective multicenter validation against clinically meaningful outcomes is needed before EBLA can support clinical decision‐making.

## 5 CONCLUSIONS

Ensemble‐Based Label Adjudication (EBLA) provides a practical framework for evaluating continuous physiological measurements against imperfect reference standards, particularly those that rely on observer‐dependent assessments. By exploiting agreement between independently fallible measurement methods, EBLA transforms the concordant subset into a high‐confidence training set for feature‐based adjudication of discordant observations.

Benchmark testing demonstrated that EBLA recovered mislabeled observations more effectively than confident learning across a range of simulated noise conditions. In the clinical application, EBLA generated revised estimates of diagnostic performance through feature‐based adjudication of discordant observations. Rather than replacing conventional validation, EBLA is intended as a sensitivity‐analysis framework that quantifies the potential impact of reference‐standard error on estimates of diagnostic performance. Its results should therefore be interpreted alongside, rather than in place of, the primary unadjusted analysis.

Future work should prospectively validate EBLA in larger multicenter cohorts, characterize the effects of correlated and structured measurement error, and extend the framework to more realistic models of reference‐standard noise.

## AUTHOR CONTRIBUTIONS

**Guillermo Gutierrez:** Conceptualization; data curation; formal analysis; methodology; supervision; validation. **Fernando Papa:** Conceptualization; formal analysis; software.

## FUNDING INFORMATION

This research received no specific grant from any funding agency in the public, commercial, or not‐for‐profit sectors.

## CONFLICT OF INTEREST STATEMENT

G.G. has applied for a U.S. patent related to the monitoring of dyspnea during mechanical ventilation. F.P. declares no competing interests relevant to this work.

## ETHICS STATEMENT

This study used only previously published, de‐identified data; therefore, institutional review board approval and informed consent were not required.

## SOCIAL MEDIA POST

New EBLA method improves validation against imperfect clinical reference standards.

## Supporting information


Data S1.

## Data Availability

All analysis code is provided as Supporting Information— The Wisconsin Breast Cancer Dataset is publicly available from the UCI Machine Learning Repository (https://archive.ics.uci.edu/dataset/17/breast+cancer+wisconsin+diagnostic). The clinical dyspnea dataset is available at Figshare (https://doi.org/10.6084/m9.figshare.31988655). Additional code repositories are detailed in the Supporting Information—S1.
