# Supplementary material for: Ensemble‐based label adjudication (EBLA): A method for validating continuous physiological measures against observer‐dependent reference standards
Source: Physiol Rep. 2026 Oct 9;14(19):e71127. doi: 10.14814/phy2.71127 (PMC13653038; doi:10.14814/phy2.71127)
Supplement: Supplementary file 1 [file PHY2-14-e71127-s001.docx]

**SUPPLEMENTARY MATERIAL**

**Ensemble-Based Label Adjudication (EBLA): A Method for Validating Continuous Physiological Measures Against Observer-Dependent Reference Standards**

***Guillermo Gutierrez, MD, PhD and Fernando Papa, BS***

***The George Washington University, Washington, DC, USA (GG)***

***Hitss Argentina, Buenos Aires, Argentina (FP)***

**Python scripts used in the EBLA analysis and primary data.**

**I. Wisconsin Breast Cancer Dataset EBLA:** [**https://doi.org/10.6084/m9.figshare.31988589**](https://doi.org/10.6084/m9.figshare.31988589)

**Proof-of-concept using the Wisconsin Breast Cancer dataset.**

1. Load data with a TRUE gold standard (pathology = M/B).

2. Create a NOISY GOLD STANDARD by randomly flipping a fraction of labels

(simulates MV-RDOS observer error).

3. Create a NOISY NEW METHOD by independently flipping a different fraction

of labels (simulates PmusPTP measurement noise).

4. Compare the two noisy instruments: identify concordant and discordant pairs.

5. Train an ensemble of ML classifiers on concordant pairs (TP + TN).

6. Apply the ensemble to discordant pairs and adjudicate by vote threshold.

7. Evaluate accuracy of the CLEANED dataset against the TRUE gold standard,

across a range of error rates.

8. Benchmark against Cleanlab (independent published method).

**II. Cleanlab comparison Python program:** [**https://doi.org/10.6084/m9.figshare.31988610**](https://doi.org/10.6084/m9.figshare.31988610)

**Self-contained Python script reproduces the Cleanlab benchmark**.

Downloads the Wisconsin Breast Cancer Dataset automatically, simulates the five error-rate scenarios, trains a Random Forest on concordant cases, passes predicted probabilities to Cleanlab's *find_label_issues()* function, and reports accuracy before and after correction. Requirements: scikit-learn, cleanlab, numpy, pandas, ucimlrepo.

**III. Application Python program for EBLA:** [**https://doi.org/10.6084/m9.figshare.31988595**](https://doi.org/10.6084/m9.figshare.31988595)

**User application that compares a new classification method against a faulty gold standard.**

It identifies discordant cases, trains a committee of ML classifiers on

concordant cases, and adjudicates discordant cases by committee vote.

Required CSV input format

Column 1 : instance ID (any string or integer)

Column 2 : new method label	(binary: any two-value encoding)

Column 3 : gold standard label (binary: same encoding)

Columns 4+: user-defined features (numeric)

**IV.** **Datafiles used in the development of EBLA:** [**https://doi.org/10.6084/m9.figshare.31988655**](https://doi.org/10.6084/m9.figshare.31988655)

**Supplementary****Figure S1**

**Supplementary****Figure S2**

peakP = Peak airway pressure.

COV = Coefficient of variation of peak pressure.

comp_DP = Computed driving pressure.

comp_C = Computed compliance.

RR = Respiratory rate.

RVI = Respiratory variability index.

TV = Tidal volume.

**Supplementary Table S1. Individual classifiers versus the majority committee.**

*Adjudication of discordant cases by each classifier alone compared with the five-member majority committee (≥3 of 5 votes), with a measure of inter-model diversity.*

***Panel A. Wisconsin benchmark — adjudication accuracy against the true (pathology-confirmed) labels, by simulated-noise scenario.***

| **Scenario (GS / NM)** | **Random Forest** | **XGBoost** | **Logistic Regression** | **SVM (RBF)** | **k-NN** | **Committee** | **Inter-model disagreement** |
| --- | --- | --- | --- | --- | --- | --- | --- |
| 10% / 10% | 0.984 | 0.981 | 0.986 | 0.984 | 0.979 | **0.982** | 3.1% |
| 15% / 15% | 0.972 | 0.968 | 0.972 | 0.970 | 0.967 | **0.968** | 1.4% |
| 20% / 15% | 0.963 | 0.960 | 0.965 | 0.960 | 0.958 | **0.960** | 1.7% |
| 20% / 20% | 0.956 | 0.953 | 0.956 | 0.951 | 0.949 | **0.954** | 2.7% |
| 30% / 20% | 0.928 | 0.924 | 0.930 | 0.930 | 0.926 | **0.935** | 4.8% |

*Values are EBLA-cleaned dataset accuracy against true labels when discordant cases are adjudicated by each classifier or by the majority committee. Committee performance approaches or exceeds the best individual model as noise increases, reaching 0.935 versus ≤0.930 at 30%/20%. Inter-model disagreement is the mean pairwise disagreement among classifiers on discordant cases. GS, simulated gold-standard error; NM, simulated new-method error.*

***Panel B. Clinical MV-RDOS / PmusPTP cohort — cross-validated AUC versus adjudication of the 54 discordant observations.***

| **Classifier** | **Cross-validated AUC** | **Adjudicated false positives** | **Specificity** | **Accuracy** |
| --- | --- | --- | --- | --- |
| Random Forest | 0.962 | 14 | 0.935 | 0.854 |
| XGBoost | 0.945 | 14 | 0.935 | 0.854 |
| Logistic Regression | 0.969 | 15 | 0.931 | 0.851 |
| SVM (RBF) | 0.928 | 9 | 0.958 | 0.872 |
| k-NN | 0.919 | 12 | 0.944 | 0.865 |
| **Committee (≥3/5)** | **—** | **13** | **0.940** | **0.858** |

*Cross-validated AUC was measured on the concordant subset; adjudication metrics were calculated after classification of the 54 discordant cases using MV-RDOS as reference. AUC did not predict adjudication performance: logistic regression had the highest AUC but the lowest specificity, whereas SVM and k-NN showed the opposite pattern. The committee provided near-median performance without prior model selection. Sensitivity was essentially unchanged (≈0.585); mean inter-model disagreement was 11.1%, and 42 of 54 cases received unanimous votes*

**Note on expected label contamination under independent errors.**

Consider two binary instruments, each with an independent probability of classification error $\varepsilon$. For any paired observation, four outcomes are possible: both instruments are correct, only the first is incorrect, only the second is incorrect, or both are incorrect. Their respective probabilities are $\left( 1-\varepsilon\right)^{2}$, $\varepsilon\left( 1-\varepsilon\right)$, $\varepsilon\left( 1-\varepsilon\right)$, and $\varepsilon^{2}$.

The two instruments are concordant when they assign the same class. Under the simple symmetric binary-error model used here, this occurs either when both are correct or when both are incorrect. The probability of concordance is therefore

$$P\left( \text{concordance} \right)=\left( 1-\varepsilon\right)^{2}+\varepsilon^{2}.$$

Among concordant observations, the only incorrectly labeled cases are those in which both instruments make the same error. Accordingly, the expected proportion of erroneous labels within the concordant subset is the conditional probability

$$P\left( \text{error}\mid\text{concordance} \right)=\frac{\varepsilon^{2}}{\left( 1-\varepsilon\right)^{2}+\varepsilon^{2}}.$$

For $\varepsilon=0.15$, the probabilities are 72.25% for both instruments being correct, 25.5% for exactly one being incorrect, and 2.25% for both being incorrect. Concordance therefore occurs in 74.5% of paired observations, and the expected contamination of the concordant subset is

$$\frac{0.0225}{0.7225+0.0225}=0.030,$$

or 3.0%. Thus, a 15% error rate in either individual instrument is reduced to an expected 3% error rate among concordant observations, representing approximately a fivefold enrichment for correct labels.

The quantity

$$2\varepsilon-\varepsilon^{2}=1-\left( 1-\varepsilon\right)^{2}$$

has a different interpretation. It is the probability that **at least one** of the two instruments is incorrect in the full set of paired observations. For $\varepsilon=0.15$, this probability is 27.75%. This value is useful for describing how often a paired observation contains any instrument error, but it is not the contamination rate of the concordant training set and should not be compared directly with the conditional error rate among concordant observations.

The distinction is important because EBLA trains its classifiers only on concordant observations. The relevant quantity for assessing training-set purity is therefore $P\left( \text{error}\mid\text{concordance} \right)$, not the probability that at least one instrument is wrong in the complete paired dataset. This enrichment depends on the assumption that instrument errors are sufficiently independent; correlated errors increase the probability that both instruments fail together and therefore reduce the purity of the concordant subset.
